# A modified formula for calculating low-density lipoprotein cholesterol values

**DOI:** 10.1186/1476-511X-9-52

**Published:** 2010-05-21

**Authors:** Yunqin Chen, Xiaojin Zhang, Baishen Pan, Xuejuan Jin, Haili Yao, Bin Chen, Yunzeng Zou, Junbo Ge, Haozhu Chen

**Affiliations:** 1 Zhongshan Hospital, Fudan University, Shanghai, China, 200032; 2 Obstetrics & Gynecology Hospital, Fudan University, Shanghai, China, 200011; 3 Children's Hospital, Fudan University, Shanghai, China, 200032

## Abstract

**Background:**

The Friedewald formula (FF) is useful for calculating serum low-density lipoprotein cholesterol (LDL-C) values, but has a remarkable deviation and limitation especially in hypertriglyceridemia. We modify the formula which is now more suitable for LDL-C calculation.

**Methods:**

2180 cases were classified into three groups according to their TG concentrations (A: < 200 mg/dl, *n *= 1220; B: 200-400 mg/dl, *n *= 480; C: 400-1000 mg/dl, *n *= 480). The concentrations of LDL-C were measured or estimated by 1) a direct measurement (DM); 2) the FF; and 3) our modified Friedewald formula (MFF): LDL-C (mg/dl) = Non-HDL-C × 90% - TG × 10%.

**Results:**

Linear regression showed a significant correlation (*P *< 0.001) between the measured and calculated LDL-C values. Bland-Altman plots indicated that the methods (DM/MFF) were in better agreement than those (DM/FF). The LDL-C/Non-HDL-C ratio in FF calculated values was significantly lower (*P *< 0.05) than that in MFF or DM values, while no significant difference between MFF and DM was found. In Group A and Group B, 4.26% and 14.79% of the MFF calculated values had more than 20% deviation from those measured by DM. These percentages were significantly lower than those calculated by FF, where 7.30% and 25.63% were observed, respectively (*P *< 0.01 and *P *< 0.001). The MFF calculated values were all positive even in Group C.

**Conclusions:**

Compared with the FF calculation, serum LDL-C values estimated by our modified formula are closer to those measured by a direct assay. The modification significantly diminishes the interference caused by hypertriglyceridemia.

## Introduction

Serum low-density lipoprotein cholesterol (LDL-C) is an independent risk factor for the development of coronary heart disease [1]. The National Cholesterol Education Program Adult Treatment Panel and other scientific societies have identified LDL-C concentrations as the primary criterion of diagnosis and treatment of patients with hyperlipidemia [2,3].

Most clinical laboratories estimated LDL-C concentrations in serum by the Friedewald formula (FF) from the concentrations of total cholesterol (TC), triglyceride (TG), and high-density lipoprotein cholesterol (HDL-C) [4-6]. The traditional FF is: LDL-C (mg/dl) = Non-HDL-C - TG/5. TG is mainly from chylomicrons and VLDL. Assuming Non-HDL-C has little or no change, if TG levels are too high, the LDL-C values would be underestimated. This could occur in the postprandial condition or patient with normal Non-HDL-C but high TG levels. The FF estimated value is not valid in specimens with TG more than 400 mg/dl [7,8]. Indeed, it has been recommended that the FF should be used with precaution in several pathologic states (diabetes, hepatopathy, nephropathy), even if the TG concentrations are between 200 mg/dl and 400 mg/dl [9,10]. In previous reports, the formula was modified to overcome the limitation [11-15]. However, these modifications were either complicated or lacking rationales. From the FF, we can see LDL-C is determined by the correlated parameters of none high-density lipoprotein cholesterol (Non-HDL-C) and TG. We may reach to a suitable point through adjusting both parameters. We proposed a modified Friedewald formula (MFF). Based on the formula, we calculated LDL-C values which were compare with the FF and a direct homogeneous assay.

## Materials and methods

Blood samples were obtained from 2180 adult outpatients, ages >18 years, at the department of clinical laboratory of Zhongshan Hospital. Blood was collected in tubes without anticoagulant from subjects after an overnight fast. The samples were allowed to clot at room temperature, and serum was obtained by centrifugation at 3000 rpm for 15 minutes. All blood lipid analyses were performed within 1 day. All subjects were classified into three groups according to the TG concentrations (A: < 200 mg/dl, *n *= 1220; B: 200-400 mg/dl, *n *= 480; C: 400-1000 mg/dl, *n *= 480). The Non-HDL-C concentrations in all samples were less than 300 mg/dl. To convert values for TG and cholesterol to millimoles per liter, we multiply the values with 0.0113 and 0.0259, respectively.

The Non-HDL-C value was estimated by the formula as follows [16]:

Lipid measurements were performed on a Hitachi 911 automatic analyzer. The LDL-C assay was performed according to Roche manufacture's specifications. At the same time, the LDL-C values were also calculated by the FF and MFF. TC and TG concentrations were determined enzymatically using CHOD-PAP and lipase/GPO/PAP methods, respectively. The HDL-C concentration was measured by phosphotungstic acid and MgCl_2 _precipitation approach. The reagents were obtained from Roche Diagnostics. The procedures and efficiency of lipid assays had been demonstrated previously [17]. The total error used in precision assessment was 3.95%-7.85% for the Roche method, as recommended by the National Cholesterol Education Program.

The FF was transformed as follows:

Multivariate linear regression analysis was used to investigate the relationship between LDL-C (expected value), TG and Non-HDL-C (explanatory variables) concentrations. Repeatability of the new formula was evaluated by Bland-Altman analysis [18]:. We compared the agreement between FF and our new formula, and calculated the mean and standard deviation of the differences (formula and lab value). The mean difference of both FF and new formula were close to zero. We concluded the MFF as follows:

Statistical analysis was performed using SPSS 11.5 for Windows (SPSS Inc., USA). Linear regression analyses were used to assess the correlations between the methods of formula calculation and direct measurement. To examine the degree of consistency between values obtained by the two methods, we used the graphical procedure outlined by Bland and Altman. Comparisons between groups were performed using the method of ANOVA. The test of Pearson chi-square was used to compare discrete variables. *P *values less than 0.05 were considered significant.

## Results

**Figure 1:**
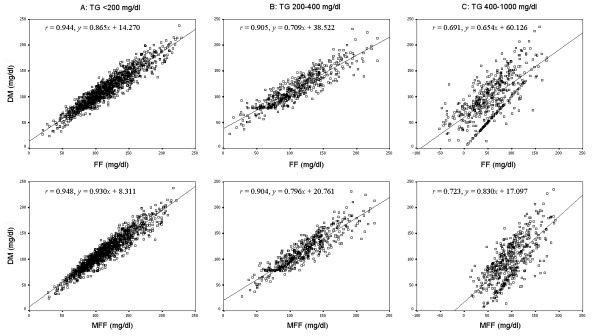
**Correlations for LDL-C in subjects with different TG values**. The LDL-C values were significantly correlated between the MFF and DM (*P *< 0.001)

**Figure 2:**
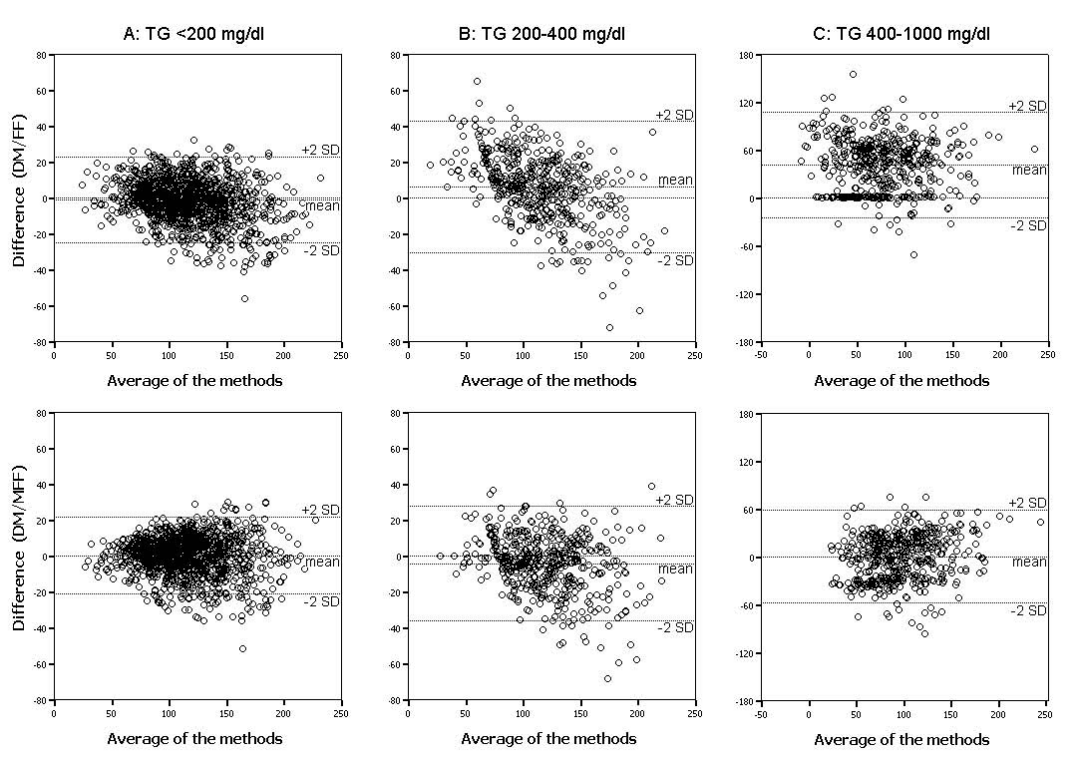
**Bland-Altman plots for LDL-C in subjects with different TG values**. In the Bland-Altman plots the difference between the direct method (DM) and the formula method for LDL-C, depending on serum TG values, was plotted against the average of the methods

**Figure 3:**
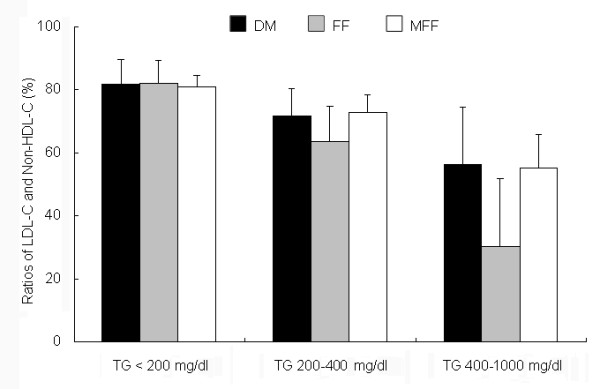
**Ratios of LDL-C to Non-HDL-C values in subjects with different TG values**. The LDL-C/Non-HDL-C ratio in FF calculated values was significantly lower in the groups with TG>200 mg/dl than that in MFF or DM values (*P *< 0.05), even in Group B (TG: 200-400 mg/dl), while no significant difference between the MFF and DM was found

As shown in the Figure 1, the LDL-C values were significantly correlated between the MFF and DM (*P *< 0.001). In the group with TG >400 mg/dl, our formula but not traditional FF fit well with the DM. As shown in the Figure 2, the difference between the DM and the formula methods for LDL-C, depending on the TG values, was plotted against the average of the methods. The Bland-Altman plots indicated a good agreement between the methods of DM and MFF. As shown in the Figure 3, the ratios of LDL-C to Non-HDL-C values were significantly decreased in the subjects with hypertriglyceridemia. This ratio in FF calculated values was significantly lower (*P *< 0.05) than that in MFF or DM values, even in Group B (TG: 200-400 mg/dl), while no significant difference between the MFF and DM was found.

In order to evaluate MFF is better than FF, we also calculated LDL-C values using MFF and FF in Group A (TG < 200 mg/dl) and Group B (TG: 200-400 mg/dl) and then compared these values with that measured by DM. We found that 4.26% and 14.79% of the values calculated by the MFF had more than 20% deviation form that measured by DM. These percentages were significantly lower than those calculated by FF, where 7.30% and 25.63% were observed, respectively (*X*^2 ^= 10.305, *P *< 0.01 and *X*^2 ^= 17.468, *P *< 0.001), suggesting MFF works better than FF in predicting LDL-C values at least in these populations. We compared LDL-C values measured by MFF or FF with those measured by DM. We found that all MFF calculated values were positive ones, while 10.42% of FF calculated values were negative. The results indicated our modified formula could provide a better estimate of LDL-C values than FF.

## Discussion

The LDL-C value is estimated using the FF, which can be transformed as follows: LDL-C (mg/dl) = Non-HDL-C - TG × 20%. In the formula, the concentration of very low-density lipoproteins (VLDL) is estimated as 20% of total TG concentrations [4]. However, the particles found in patients with hypertriglyceridemia are usually a heterogeneous mixture of chylomicron remnants, VLDL, and VLDL remnants [19]. It is well known that the ratio of TG to cholesterol (TG/cholesterol) varies a lot within these particles. When the TG concentration is more than 400 mg/dl, the cholesterol in TG-rich lipoproteins is overestimated by the FF method, resulting that the calculated LDL-C value even appears negative [20]. Therefore, this method has a limitation in clinical application.

In this study, the FF estimated value was calibrated using different coefficients to avoid LDL-C underestimation. We examined the correlation between traditional FF and our new formula, and found that both of FF and MFF fit well with the DM in the subjects with TG < 400 mg/dl. While If the TG concentrations were higher than 400 mg/dl, our formula but not traditional FF fit well with the DM. However, because high coefficients do not necessarily mean that two methods agree. We assessed the degree of agreement between the two methods using the Bland-Altman graphical technique. The Bland-Altman graphs are plots of the difference between the two methods against their mean. The degree of agreement is indicated by calculating the bias, estimated by the mean and SD of the differences. The figure 2 showed an obvious relationship between the differences and the mean. The Bland-Altman plots suggest that the methods (DM/MFF) are in better agreement than those (DM/FF). Moreover, The MFF calculated values had a smaller deviation from the DM values and a regular relationship with the Non-HDL-C values, showing our new formula works better than classical FF.

In summary, a modified formula for LDL-C calculation was designed in this study: LDL-C (mg/dl) = Non-HDL-C × 90% - TG × 10%. The MFF estimated LDL-C values have following characteristics: 1). They are closer to those measured by the DM than those estimated by the FF; 2). They have a stable LDL-C/Non-HDL-C ratio; and 3). The interference caused by hypertriglyceridemia might be significantly diminished. Therefore, the MFF has higher accuracy than FF. Although our findings need to be confirmed in additional studies, they hold a promise of broadening the usage of the FF in LDL-C measurement.

## Competing interests

The authors declare that they have no competing interests.

## Authors' contributions

YC and XZ carried out the data analysis, provided the hypothesis and drafted the manuscript. BP carried out clinical assays of blood lipids. XJ performed the statistical analysis. HY and BC collected and managed the blood samples. YZ, JG and HC conceived of the study, and participated in its design and coordination.

All authors read and approved the final manuscript.
